# ADVANCED CARE PLANNING: LEVERAGING PARTNERSHIPS TO IMPROVE STATE-WIDE DISSEMINATION

**DOI:** 10.1093/geroni/igad104.2388

**Published:** 2023-12-21

**Authors:** Lisa Bodenheimer, Jennifer Rutberg, Pat Tadel, Jennifer DeGennaro

**Affiliations:** Rowan University, Stratford, New Jersey, United States; NJ Department of Human Services, Division of Aging Services, Trenton, New Jersey, United States; Respecting Choices, Oregon, Wisconsin, United States; Rowan-Virtua SOM / NJISA, Stratford, New Jersey, United States

## Abstract

Rowan-Virtua School of Osteopathic Medicine, The New Jersey Institute for Successful Aging (NJISA), in collaboration with the New Jersey Division of Aging Services (DoAS) and Respecting Choices, implemented a state-wide project training workforce professionals in Advanced Care Planning (ACP) conversations with older adults. The concept: proactively leverage the long-term care management relationships in community-based, high-touch programs. NJISA identified Respecting Choices, an evidenced-based program approved by the Administration for Community Living. Respecting Choices offers standardized certification for ACP facilitators and instructors, paving a pathway for workforce sustainability. To achieve state-wide penetration, NJISA partnered with NJ DoAS to target staff and professionals serving the state aging network. NJ DoAS provided access to county-level case managers serving the Jersey Assistance for Community Caregiving (JACC) program, Program of All-Inclusive Care for the Elderly (PACE) nurses and social workers, and NJ DoAS staff. Also included in training efforts were staff from select senior affordable housing sites. With funding support through the New Jersey Health Foundation, NJISA and Respecting Choices offered virtual, live training course for certification of First Steps ACP facilitators and instructors and Design and Implementation for state aging networks, and ACP Instructors. NJ DoAS modified the electronic documentation system utilized by JACC staff to capture on-going ACP conversations and outcomes. Despite barriers and mid-course corrections, training totals include (30) certified ACP Facilitators, (3) leaders for Design and Implementation, and (5) certified ACP Instructors. Response to curriculum was positive; participants indicating greater confidence navigating ACP conversations. Future plans to continue dissemination have been discussed.

